# Notes and News

**Published:** 1900-10-27

**Authors:** 


### Notes and News.

The foundation of the New Croydon and Wimbledon Joint Small-pox Hospital has been laid at North Cheam. It will consist of three ward blocks for forty-eight patients, and the usual offices, administrative and other buildings. It is to cost upwards of £23,000.

The building of the new infirmary at Newcastle is at a standstill. The difficulty, it is stated, arises from the fact that the funds are not actually in hand, and Mr. John Hall's bequest of £100,000 has not yet been paid in. The obstacles appear to be of a technical and legal nature, which should be removed before very long.

The authorities at the Convalescent Home for the Household Brigade at Golder's Hill have set a good example by permitting the inmates to learn a light and interesting employment to relieve the tedium of convalescence. The Brabazon Employment Society and the Soldiers' and Sailors' Help Society have combined to provide a teacher. The cobbler's art is also to be taught if it can be arranged.

At the Conference of Delegates of the London Poor Law Guardians on the question of Sanatoria for Consumptive London Paupers, held last week at St. Martin's Town Hall; it was after some debate decided to approach the Local Government Board with the view to providing accommodation. A deputation of six medical men was appointed to represent the guardians. At the conference, Sir William Broadbent spoke, and advocated the notification of consumption. The whole meeting was in favour of the establishment of Sanatoria.

A most unwise proposal was recently put forward at a meeting of the Swansea Hospital Managers. This proposal was to issue a form to be filled up by each patient on leaving, stating his experience in the hospital. Although the majority present did not approve of the suggestion, no one made the strong protest we should have expected. Any patient having a complaint to make can approach the Board of Management, and most would hesitate to do so for a trivial reason, but the difficulties arising from the careful investigation of every small complaint would be endless, for all complaints would either have to be investigated, or the record of them would be a mere farce. The matter has been left to the House Committee to decide, though it came through them to the managers.

The Metropolitan Asylums Board is about to erect another small-pox hospital, to be called the Joyce Green Hospital. They will ask a loan of £75,000 from the London County Council for the purpose.

Dr. Koch and his assistant, Dr. Olwig, have arrived at Marseilles from Egypt, where Dr. Koch has been studying the bacillus of African fever. He states himself as highly satisfied with the results of his investigations.

A course of eight lectures will be delivered at the Hospital for Diseases of the Skin, Blackfriars, on Tuesdays, at 5 p.m., commencing on Tuesday, October 23rd, by Dr. Phineas S. Abraham, Mr. George Pernet, and Dr. C. F. Marshall.

A new hospital is to be erected for the use of the inhabitants of Bo'ness, in Scotland. The first proposal embodied a scheme for a joint hospital for Bo'ness and Grangemouth; but, as the powers of the two localities were divided as to the allocation of the accommodation, Bo' ness has decided to act independently.

The East Suffolk and Ipswich Hospital has received a donation of £1,000 to endow a bed in memory of William and Rosa Turner. The gift comes from the ten children of Mr. and Mrs. Turner, each having given £100 for the purpose. The Ipswich Hospital is fortunate in recording the memory of nine other persons in the same manner.

The Public Analyst for the City of Manchester reports that he analysed 460 articles of food, and amongst them only 9 were found by him to be adulterated. As many as 263 samples of milk were examined, and all but one were pronounced genuine. The percentage of genuine butter was not quite so good, but the whole report must be regarded as most satisfactory.

The hospitals are frequently used as a bait to secure money. One of the most recent examples of this is the case of a journalist who evolved a scheme to publish a magazine, the profits of which he stated were to go to the hospitals. The scheme went no further than obtaining money from guarantors on the understanding that they were to be employed in the production of the magazine.

The Board of the National Hospital for the Paralysed and Epileptic have passed the following resolution:—"That in view of the allegations in the public Press injurious to the interests of the hospital, it is desirable that an inquiry should be forthwith held into the charges formulated by the medical staff against the domestic condition and management of the hospital, and that the Board should request some independent legal authority to conduct such an inquiry." The work of the hospital is proceeding as usual.

The outbreak of rabies reported in South Wales has brought forward an attack upon Mr. Long and his methods by the National Canine Defence League. The Society probably fears a return to the muzzling order. If statistics are worth anything there can be no doubt that at the end of the period when muzzling had been resorted to rabies was practically non-existent. Nevertheless, we feel the objection to muzzling in one district and not in another is one that is universally admitted, and it is not to be supposed Mr. Long will advocate such a measure again.

